# Laterality in Children: Evidence for Task-Dependent Lateralization of Motor Functions

**DOI:** 10.3390/ijerph17186705

**Published:** 2020-09-15

**Authors:** Danilo Bondi, Giulia Prete, Gianluca Malatesta, Claudio Robazza

**Affiliations:** 1 Department of Neuroscience, Imaging and Clinical Sciences, University “G. d’Annunzio” of Chieti-Pescara, 66100 Chieti, Italy; 2 Department of Psychological, Health and Territorial Sciences, University “G. d’Annunzio” of Chieti-Pescara, 66100 Chieti, Italy; giulia.prete@unich.it (G.P.); gianluca.malatesta@unich.it (G.M.); 3 Department of Medicine and Aging Sciences, University “G. d’Annunzio” of Chieti-Pescara, 66100 Chieti, Italy; c.robazza@unich.it

**Keywords:** handedness, lateralization, asymmetry, fine motor skills

## Abstract

The behavioral preference for the use of one side of the body starts from pre-natal life and prompt humans to develop motor asymmetries. The type of motor task completed influences those functional asymmetries. However, there is no real consensus on the occurrence of handedness during developmental ages. Therefore, we aimed to determine which motor asymmetries emerged differently during childhood. A total sample of 381 children in grades 1 to 5 (6–11 years old) of primary school were recruited and tested for two fine coordination tasks (*Floppy*, led by dexterity, and *Thumb*, led by speed-dominated skills) and handgrip strength (*HS*). Data about their handedness, footedness and sports participation were also collected. Children performed better with their dominant side, especially for the *Floppy* and *HS* tests. The asymmetries were more marked in right-handed children and did not differ by age, gender or type of sport. Our findings support the thesis of a functional lateralization in complex coordinative tasks and in maximal strength during developmental ages. Furthermore, our findings extend the evidence of a stronger lateralization in right-handed individuals, demonstrating it at a functional level in primary school children performing motor tasks. Fine motor skills allow a “fine” understanding of developmental trajectories of lateralized behavior.

## 1. Introduction

Besides the apparent physical symmetry, many species including humans are greatly asymmetric, both at a structural and functional level [1,2]. The most evident functional asymmetry in the nervous system concerns handedness, and it corresponds to the preference for the use of the right rather than left side of the body (mainly hand and foot), which is present in almost 90% of the overall population (for a review, see [3]). This population-level asymmetry seems to be mostly independent of demographic features, such as gender and ethnicity, and it has been found to be present even in pre-natal life [4,5,6]. Recently, a large-scale investigation of early life factors on hand preference concluded that, despite some tiny predictive effect, the biological bases are still largely unexplained, and that hand preference is not a heritable trait [7]. The evidence according to which older adults reveal a stronger laterality preference compared to younger adults [8] confirms that handedness develops—or, at least, becomes increasingly more stable—in post-natal life, and remains consistent during adulthood [9].

The centrality of this topic in the physical, physiological and neuropsychological frame is well defined considering the amount of standardized and “home-made” tests available for investigating handedness [10]. The majority of these measures are based on self-report assessments (e.g., “do you write with your left or right hand?”), but also more sophisticated tests have been developed, and some of them are specific for children (for a review, see [11]).

At a neurophysiological level, the behavioral preference for the use of one side of the body corresponds to a contralateral hemispheric superiority in motor control, due to the crossed motor (and sensory) nervous pathways in humans [12]. This means that if a person is strongly right-lateralized (right-hander), her/his left hemisphere is more competent than the right hemisphere either in fine motor control, or in strength, or in both of them. Thus, behavioral laterality corresponds to a cerebral imbalance, which would be advantageous, allowing each side of the brain to develop its specific competencies.

The great attention focused on this issue is not surprising if one considers that some theories (and evidence) suggest a relationship among hemispheric asymmetries, their specific direction and some cognitive and mental skills. It has been shown, for instance, that a less lateralized brain, which would correspond to a less lateralized behavioral preference, is related to some deficits in cognitive development [13] and to a higher risk of mental health [14,15,16], also in children [17]. A higher prevalence of left or mixed handedness has been reported in several developmental disorders, both in the socio-communicative domain (e.g., autism spectrum disorders; for a review, see [18]) and in the motor domain (e.g., developmental coordination disorder; for a review, see [19]), compared to the general population. This evidence is consistent with the hypothesis that atypical cerebral and/or behavioral lateralization may reflect a potentially dysfunctional brain organization, possibly due to the non-optimal distribution across the two hemispheres of specific functions. As a result, a possible role of an atypical lateralization during early mother–infant interactions has been very recently suggested [20,21].

Starting from these premises, a crucial issue in this field is the understanding of the time course of handedness expression. The investigation of the specific age in which this lateralized bias becomes stable covers a crucial role from different perspectives. From one hand, the role of the environment as opposed to that of genetic heritage has been partially explained by the evidence of laterality biases even prenatally [4,5,6,22]. On the other hand, understanding the evolutionary root of such a key component of adult life is crucial in order to try to understand whether the laterality preference established in childhood remains stable or it undergoes some developmental changes over time. Although many studies have been conducted and many hypotheses have been advanced to account for this phenomenon, there is no real consensus on when and how handedness determination occurs in ontogeny. This is probably due to the large quantity of assessment methods used for behavioral asymmetries in manual skills both in children and in adults, often representing a confounding variable causing inconsistent results [11]. Evidence showed that a right hand preference can be observed rather reliably in babies by 7 months of age during object manipulation [23] and by 11 months of age in reach-to-eat behaviors [24]. Moreover, by 2 years of age, a role-differentiated bimanual manipulation emerges [25]. However, it is generally accepted that a fairly accurate identification of the direction of hand dominance can be preliminarily assessed at the age of 3 years [26,27].

A further important issue in this frame is that, besides left/right direction, many studies have focused on the degree of hand preference and on the difference in performance (e.g., strength, speed, precision) between the dominant and non-dominant hand. In particular, it was found that the difference in terms of performance between the two hands is larger (in favor of the dominant hand) in 3-to-6-year-old children compared to older children [28], with left-handed children showing a smaller difference compared to right-handed children [29]. Therefore, hand preference and hand performance for unimanual tasks seem to increase with age and tend to manifest more evidently in right-handed children, possibly at the same pace as the development of brain functions and as the increase in experience in motor activity (Fennell et al. [30]; for a review, see Scharoun and Bryden [11]).

A further crucial point in this domain is that hand preference is associated with motor skills: the difference in hand grip strength in favor of the dominant hand is known as the 10% rule [31]. Hepping and colleagues [32] tested this issue in children and adolescents and demonstrated this asymmetry in right-handed but not in left-handed individuals. Beyond strength, functional asymmetries in the early phase of learning are more evident in right-handed individuals [33]. Regarding age stages, pre-school children (particularly the left-handed ones) have a weak hand preference and, throughout the transition to older ages (10 years old), they undergo a period of motor skills refinement while increasing their reliance on the preferred hand [11].

Starting from these premises, it is reasonable to hypothesize that hand preference—and its behavioral expression in the developmental trajectories of brain lateralization—is strictly associated with the maturation of fine motor coordination [34]. In a recent definition of fine motor skills, three clusters were proposed: (1) grapho-motricity, which integrates visual and motor inputs when performing small-scale hand movements, often through a graphic tool; (2) dexterity, which encompasses performances obtained by manipulating small objects; and (3) speed-dominated skills, which encompass rapid and simple movements of fingers or hands [35]. The understanding of these diverse fine motor skills in relation to laterality may improve the comprehension of specific developmental trajectories [36]. In fact, the type of motor task completed influences asymmetries in young ages: lateral asymmetry has been proved to be task-specific both in pre-school [37] and in early school age [38]. However, since few (and conflicting; e.g., [39]) results have been described in this area, further investigations examining such a relationship are needed.

More generally, laterality preference is associated with one’s success in sports. Specifically, the possible influence of sports and which kind of sport activities are mainly practiced by children is an important topic in this frame, considering the general evidence according to which ambidexterity (i.e., non-lateralized hand preference) is more common in athletes than in the general population [40,41]. For instance, in a sample of wrestler children, right-handed—but not right-footed—ones performed faster in a multiple sprint task compared to left-handed ones [42]. An over-representation of left-sided athletes has been reported for handball in young age [43], and a higher probability of winning medals has been reported for under-15 judokas [44]. The advantage of left-sided athletes in sports has been attributed to the scarcity of practice strategies against left-orientation [45]. To be noted, this left-sided over-representation of athletes often refers to open-skill-dominated sports, which require continuous adaptations of motor behaviors to an unpredictable environment, rather than closed-skill-dominated sports characterized by self-paced movement patterns in a predictable environment [46]. Summarizing, sports are full of lateralized behaviors, and the role of laterality in talent identification and athlete development is worth investigating [47].

In a preliminary study with children in the second grade of primary school, it was demonstrated that functional asymmetry is more likely to emerge in complex fine coordinative tasks [38]. In the current study, we aimed to extend this prior research by recruiting a larger sample and covering the whole age window of primary school. In addition, we wanted to examine functional asymmetries in children by hand preferences (left-handed vs. right-handed). We also aimed at defining the developmental trajectory of lateralization during childhood, starting from the hypothesis that a strengthening of dominant hand preference concerning fine motor skills occurs from 6 to 11 years of age.

## 2. Materials and Methods

### 2.1. Design and Participants

In the current cross-sectional study, children were assessed at their school in the presence of their teachers and physical education experts, during regular physical education (PE) lessons, as an implementation of a testing package included into a physical education project in primary school. To recruit the participants, we first looked for available schools in the Abruzzo region, Italy; secondly, we planned activities together with teachers. Measurements were conducted 4 to 7 months from the beginning of the school year. The study ethics conformed to the Declaration of Helsinki and was approved by the Health Department of the Abruzzo Region in reference to the Regional Prevention Plan 2014–2018—Program 2, Action 2. Permissions were obtained by the school administrations and informed consent was signed by the parents of the children.

A total sample of 381 children in grades 1 (6–7 years old), 2 (7–8 years old), 4 (9–10 years old) and 5 (10–11 years old) living in the Abruzzo region, Italy, were recruited from 7 local public schools. Eleven participants with certified disability were included in the testing activities but excluded from the data analysis. The sample for data analysis was composed of 370 healthy children, of whom 101 were from 1st grade (56 girls and 45 boys), 105 from 2nd grade (48 girls and 57 boys), 91 from 4th grade (55 girls and 36 boys) and 73 from 5th grade (41 girls and 32 boys). Sport participation was considered if frequency was higher than twice a week (including match and trainings) and participants were involved in sports activities for at least 0.5 years, in the framework of national sports federation or national sports promotion bodies. According to the definition of open-skill and closed-skill sports by Nuri and colleagues (2013), and taking into account a minimum time of participation of 0.5 years, 46% of participants were involved in closed-skill sports and 36% in open-skill sports, whereas 18% of participants did not participate in any sports. Results about anthropometrics and motor performances are reported elsewhere. In brief, the averages of body mass index, waist-to height ratio, long jump, 4 × 10 shuttle run test (4 × 10 SR) and standing long jump (LJ) were: 18.82 ± 3.38 Kg/m^2^, 0.48 ± 0.05, 1.16 ± 0.21 m and 14.67 ± 1.66 s, respectively (for the analyses, see [48]).

### 2.2. Procedures

Participants maintained their regular training schedule throughout the testing period. Measurements were conducted in the school gyms, in a comfortable and safe environment. Days and time of day were related to the typical PE schedule of each class. In a first step of the procedure, participants were asked to report their left or right hand and foot preference, concerning specific everyday life activities, such as writing, punching and kicking. Then, two fine coordination tests were used, in addition to a test measuring maximal strength. Participants received specific instructions on how to complete the tests and were not allowed to see the tests of their schoolmates. Specifically, *Floppy* is a transitive (tool-related) test which requires participants to insert fourteen floppy disks one at a time in the proper case with one hand, as fast as possible, while the other hand controls the case. This test was not used before; the test–retest reliability in early school age yielded an ICC coefficient of 0.88 (unpublished data). *Thumb* is an intransitive test that requires participants to touch each finger of one hand with the thumb of the same hand in an alternating pattern (2nd, 3rd, 4th and 5th finger, and reverse), as fast as possible. This test was based on the visual-motor control assessment of the BOT-2 battery [49]; the test–retest reliability in early school age yielded an ICC coefficient of 0.93 (unpublished data). Time of completing *Floppy* and *Thumb* with each hand was measured with a stopwatch. According to the abovementioned definition [35], the *Floppy* test measured dexterity, and the *Thumb* test measured speed-dominated skills. For the *Handgrip Strength (HS)* test, according to the ALPHA health-related fitness test battery for children [50], children remained standing and performed their maximal grip strength keeping the arm straight at their side. The test was carried out three times with a handgrip dynamometer (T.K.K. 5101 GRIP-D, Takei Scientific Instrument, Niigata, Japan) and the best performance was recorded. Participants completed all the tests with both hands. The order of execution, among the dominant and non-dominant sides, was randomized among participants, who were tested individually. As reported above, the whole packages of tests included also the 4 × 10 m shuttle run test (4 × 10 SR) and the standing long jump test (LJ). The first test measures speed and agility and the second one measures the functional strength of the lower limbs [50]. The sequence of the tests was randomized among participants. Warm-up was done before the tests, leaving teachers conducting this phase as they planned. Concerning handgrip strength and long jumps, at least 1 min of rest was provided between the trials.

### 2.3. Data Analysis

Asymmetry indexes (*Asy-RATIOs*) of the three tests were calculated as percentage variation of the non-dominant side performance. Next, the *Asy-RATIO* of HS was inverted with respect to the other two indexes, so that in the HS test a negative ratio indicated a worse performance of the non-dominant side, and a cumulative index of asymmetry (*Asy-INDEX*) was calculated averaging the three *Asy-RATIOs*.

Statistical analyses were carried out using the R-based open-source software Jamovi Version 1.2.5.0 (retrieved from https://www.jamovi.org) (Jamovi project, Sidney, Australia), using the Flexplot module for building the graphs. Prevalence of hand and foot self-assessed preference correspondence was tested with the χ^2^ test of association, clustering by school grade, type of sport and side preference. Assumption checks conducted before all the following tests included the Shapiro–Wilk test for normality and, regarding the ANOVAs, Levene’s test for homoscedasticity. The non-parametric Wilcoxon *t*-test for paired samples was used to compare the manual performances of dominant and non-dominant hands. The *Asy-INDEX* was compared by school grade, gender and type of sport separately using one-way analyses of variance (ANOVAs), and by manual preference with a Student’s *t*-test for independent samples. Independent variable interactions were not examined due to the unbalanced number of participants in the sub-groups. Both the *Floppy* and *Thumb* tests were log_10_-transformed for normalization. Next, a repeated measures ANOVA, type III sum of squares, was used to compare dominant vs. non-dominant hand as a within-subject factor, and school grade and side preference as between-subject factors. All the results (the three tests herein presented, and the others reported elsewhere, as part of the same testing package) were analyzed with a correlation matrix (non-parametric Spearman method), stratifying by side preference.

## 3. Results

The whole data set is available as Supplementary Materials. As reported elsewhere, the *HS* of our participants was higher than the European reference value in early school-aged children, but lower in older children [48]. References were not available for the *Floppy* and *Thumb* tests. The self-reported laterality preference for hand and foot showed that 41 participants (10.8%) were left-handed. The correspondence between the side preference between hand and foot (i.e., right hand and right foot preference) was found in 88.1% of participants. The correspondence between hand and foot preferences varied with school grade, *χ*^2^(3) = 8.337, *p* = 0.040, even if the trend was not linear: the correspondence was found for 91.7% of participants in the first grade, for 78.9% in the second, 92.1% in the fourth and 87.3% in the fifth. A tendency was found by gender (90.8% in males, and 85.0% in females, *χ*^2^(1) = 2.641, *p* = 0.104). Regarding the type of sport, the greatest correspondence was found for children involved in closed-skill sports compared to open-skill sports or no sport groups (93.3%, 84.2% and 83.9%, respectively, *χ*^2^(2) = 6.818, *p* = 0.033). Right-handed children showed a significantly greater prevalence of correspondence compared to left-handed ones (90.5% vs. 66.7%, *χ*^2^(1) = 16.012, *p* < 0.001).

Comparing the dominant vs. non-dominant side, a significant difference emerged in favor of the dominant hand (*p* < 0.001 for both the *HS* and *Floppy* tests, *p* = 0.028 for the *Thumb* test). However, the effect size was medium and small for the *Thumb* test and the *HS* test (Cohen’s *d* = 0.689 and 0.388, respectively), and it was trivial for the *Thumb* test (Cohen’s *d* = 0.090). Indeed, the greatest *Asy-RATIO* was found for the *Floppy* test (non-dominant side performed worse, with a median of 12.01% of increment in the time of completing), followed by the *HS* test (non-dominant side performed worse, with a median of 3.45% of decrement in the maximal strength) and *Thumb* test (non-dominant side performed worse, with a median of 2.08% of increment in the time of completing). *Asy-INDEX* did not differ by age (50th percentiles: 6.54% in the first grade, 5.51% in the second, 5.42% in the fourth and 6.67% in the fifth), gender or type of sport. A difference in manual preference was observed, with right-handed children (50th percentile: 6.74%) more asymmetric (*p* = 0.002, Cohen’s *d* = 0.596) than left-handed ones (50th percentile: 1.09%).

**Figure 1:**
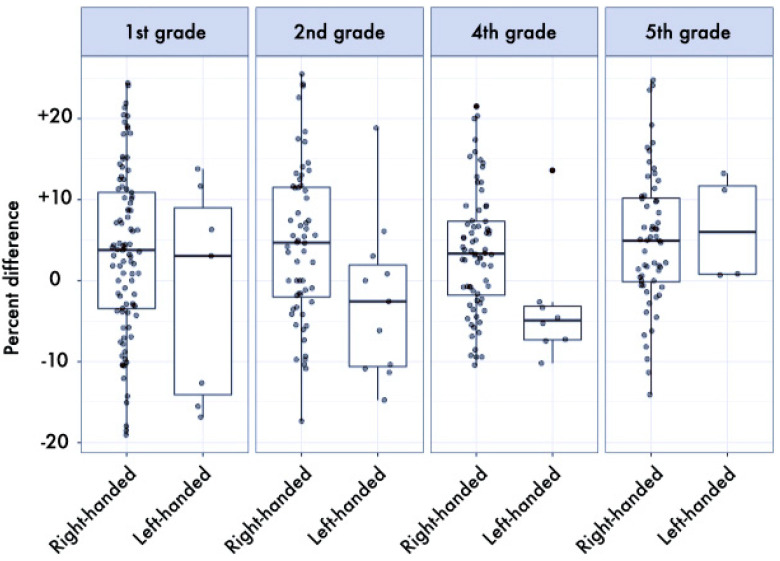
Boxplot (median and interquartile range, and individual values) of percent differences between dominant and non-dominant hand in the *HS* test stratified by school grade and hand preference. Values above 0% indicate better performance of the dominant hand. The median of the asymmetry index in favor of the dominant side was 2.63% for left-handed and 3.85% for right-handed children.

**Figure 2:**
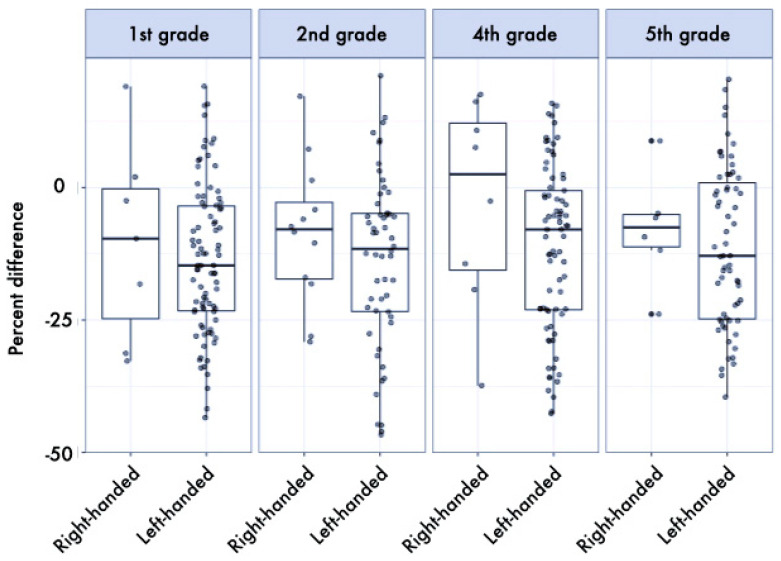
Boxplot (median and interquartile range, and individual values) of percent differences between dominant and non-dominant hand in the *Floppy* test stratified by school grade and hand preference. Values below 0% indicate better performance of the dominant hand. The median of the asymmetry index in favor of the dominant side was 7.37% for left-handed and 12.56% for right-handed children.

**Figure 3:**
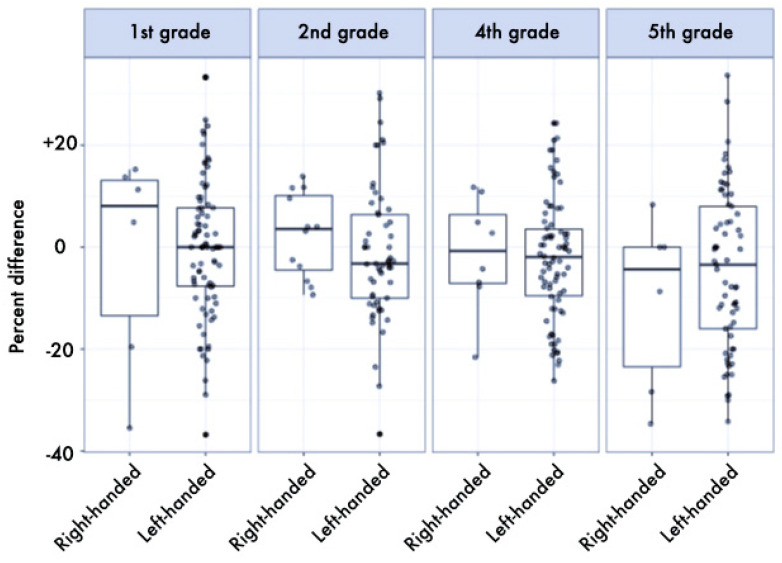
Boxplot (median and interquartile range, and individual values) of percent differences between dominant and non-dominant hand in the *Thumb* test stratified by school grade and hand preference. Values below 0% indicate better performance of the dominant hand. The median of the asymmetry index in favor of the dominant side was 1.39% for left-handed and 2.56% for right-handed individuals.

The results of the ANOVAs showed a significant difference by dominance, in favor of the dominant hand, for both the *Floppy* and *HS* tests (*p* < 0.001, *η_p_*^2^ = 0.099 and *p* = 0.028, *η_p_*^2^ = 0.016, respectively). Left-handed children were slightly faster in the *Thumb* test than right-handed ones, while right-handed children were slightly stronger than left-handed ones, even if not reaching statistical significance (*p* = 0.066, *η_p_*^2^ = 0.011, and *p* = 0.160, *η_p_*^2^ = 0.006, respectively). In all tests, as expected, the effect of age stage was significant, with older children performing better (*HS*: *p* < 0.001, *η_p_*^2^ = 0.232, *Floppy*: *p* < 0.001, *η_p_*^2^ = 0.220, *Thumb*: *p* < 0.001, *η_p_*^2^ = 0.233) (a more detailed description of this result is provided in [48]). The asymmetry was more marked in right-handed children, as demonstrated by the interaction dominance × hand preference, significantly for the *HS* test (*p* = 0.020, *η_p_*^2^ = 0.017, see Table 1 and Figure 1) and as a tendency for the *Floppy* test (*p* = 0.086, *η_p_*^2^ = 0.009, see Table 2 and Figure 2), whereas no difference was found for the *Thumb* test (see Table 3 and Figure 3). Finally, a trend was also found in the interaction school grade × hand preference, even if not reaching statistical significance (*p* = 0.175, *η_p_*^2^ = 0.016): right-handed children were weaker in the first grade, but far stronger in the fifth grade, in respect to left-handed. No other differences were found.

Table 4 shows the correlation results: according to the absolute criteria [51], the three manual tasks showed a moderate correlation in right-handed children and a low correlation in left-handed children. To be noted, the differences in sample size affected the *p* values but not the correlation coefficients [51]. In addition, there were some low or moderate correlations of the three manual tests with the other fitness tests, namely the 4 × 10 SR and the LJ tests, both in left-handed and right-handed individuals.

## 4. Discussion

We hypothesized that motor asymmetries emerged differently with respect to the type of manual task, led by strength, dexterity or speed-dominated skills [35], in primary school-aged children. First of all, the results of the present study showed that the prevalence of left-handers (10.9%) in children from 6 to 11 years of age was in line with the adult population percentage [52]. Furthermore, we also aimed to examine the trajectory of functional lateralization in childhood. Lateralized behaviors are complex individual features that emerge from developmental psychobiological pathways [53]. In particular, handedness, and the right-handed prevalence, start from pre-natal life, as confirmed by analyzing the kinematics of upper limbs movements [22]. The sensory-motor requirements prompt humans to develop a motor asymmetry based on a side dominance which, once established, remains consistent throughout the lifespan [9,22].

Right-handers have a more pronounced hemispheric lateralization for cognitive functions, relying more on asymmetries between hemispheres, whereas left-handers are more likely to demonstrate an atypical lateralization [54]. This more pronounced lateralization for right-handers occurs also at a functional level, as left-handers typically exhibit weaker preferred hand tendencies than the counterparts [11]. Concerning strength, this difference is known as the 10% rule in favor of the preferred hand [31], and it has been demonstrated that in developmental ages, this rule accounts only for right-handed individuals [32]. Concerning motor control, differences in the cortical sensorimotor network have been found while performing motor tasks with the dominant or non-dominant hand [55] and functional asymmetries were found to be more evident in right-handed individuals [33]. In this framework, task complexity significantly accounts for hand selection [9], as asymmetries have been proven to be task-specific in pre-school and early school ages [37,38].

Our findings support the thesis of a more pronounced functional lateralization in complex coordinative tasks and in maximal strength, as investigated by means of different tasks. In particular, the *Floppy* test can be considered as a complex task, requiring role-differentiated bimanual manipulation, based on dexterity and with an increasing difficulty while performing. The *Thumb* test can be considered a simpler task than the *Floppy* test based on speed-dominated skill, without the need of a strategy to accomplish the goal. The *Floppy* test revealed a higher asymmetry in favor of the dominant side, followed by the *HS* test. In contrast, the *Thumb* difference comparing the dominant and non-dominant side was only trivial. Therefore, we demonstrated that motor asymmetries in fine coordinative tasks are task-dependent (or complexity-dependent) over the developmental age of children. The amount of asymmetry in our sample was higher than the 10% rule [31] for the *Floppy* test (50th percentile: 12.01%) and lower for the *HS* test (50th percentile: 3.45%) and *Thumb* test (50th percentile: 2.08%). All in all, the median of the *Asy-Index* including the three tasks was 6.03% in favor of the dominant hand. The age stages did not significantly influence these asymmetries.

In agreement with Hepping et al. [32], who examined healthy individuals aged 4 to 17 years, we found that the asymmetry in handgrip strength occurred only in right-handed individuals. In addition, we observed a strong tendency for right-handed individuals to be more asymmetric in the *Floppy* test compared to their left-handed counterparts. Such difference did not emerge for the *Thumb* test. Therefore, we found support of a more pronounced functional lateralization in right-handed individuals performing manual tasks led by strength or complex fine motor skills.

Manual strength and manual fine motor skills follow similar developmental trajectories throughout the primary school ages [48]. Our results revealed higher coefficients of correlations among the three manual tasks than with the other fitness measures. Differences in three manual tasks were observed between right-handed and left-handed children, with the former showing higher correlation coefficients. Considering the three manual tasks as a whole cluster, the median of the manual *Asy-Index* was found to be 6.03% in favor of the dominant hand. Again, this index was significantly higher for right-handed children (6.74%) than for left-handers (1.09%). The human motor system is a network of interacting sub-components [56], with complex relations between fine and gross motor functions in the developmental age [57]. In this view, further studies should take into account different coordinative tests leading to a deep understanding of the functional asymmetries throughout developmental ages, examining the more prominent lateralization of motor functions in right-handed individuals in a wide range of tasks. Those studies may find the best tests for identifying some functional or pathological limitations, as already proposed for older ages [58]. In this frame, some novel insights may help to deal with developmental coordination disorder, for example, to examine whether a more prominent lateralization of motor functions, as common in right-handed children, would decrease the motor learning and performance issues typical of this disorder [59].

We did not find significant performance differences when comparing right-handed vs. left-handed children, although trivial tendencies were observed for left-handers, being slightly faster in the *Thumb* test, and right-handers being slightly stronger in the *HS* test. Regarding the latter result, a trend was found for right-handed children who outperformed left-handed peers in older, but not younger, ages. Considering the topic of talent identification and athlete development related to lateralization (for an overview of the topic, see [47]), further investigations are needed to probe whether or not side preference influences the development of sports performances. Footedness should also be taken into account, as it has been demonstrated to be a better predictor than handedness of sports performance [60]. Those studies should consider the multifaceted process of lateralization in the framework of biosocial developmental trajectories [53], taking into account motor learning, physical performances, practice strategies and context-based advantages.

Crossed laterality is the phenomenon of individuals who do not exhibit hand, eye, foot or ear dominance consistently as right- or left-sided. Despite some initial findings, it has been affirmed that this phenomenon does not entail impairments in academic achievement or intelligence [61]. However, the consequences for motor functions have not been extensively studied. In our sample, right-handed participants exhibited a crossed hand–foot dominance less frequently than left-handed peers. This result does not agree with a large-scale study conducted on Turkish people [62]. Finally, considering the type of sports (i.e., closed-skills and open-skills), children who practiced closed-skill sports revealed a lower incidence of crossed dominance. Thus, considering the discrepancy in our findings compared to the cited one, further studies investigating differences by type of sports, as well as by age, ethnicity and gender are worth considering.

It is worth noting that our results cannot be ascribed to better visuomotor adaptations due to a fine coordinative specific training on dominant hand [63], as the fine motor tasks we used were unfamiliar to participants. An interesting perspective may emerge when investigating the role of differentiated bimanual manipulation during developmental ages [64], analyzing spatio-temporal processes [65] while performing simple and complex fine motor tasks

## 5. Conclusions

This work dealt with the lateralization of motor functions in developmental ages, showing that functional asymmetries are more prominent in a complex, rather than simple, task of fine motor skills. The asymmetry in favor of the dominant hand was also confirmed for the handgrip strength.

Right-handed children exhibited a more prominent asymmetry in both tasks compared to left-handers. This result extends the findings of a stronger lateralization in right-handed individuals, demonstrating a functional lateralization in primary school children when performing motor tasks. Novel insights on kinematic processes during specific motor tasks will help to extend the current findings on neuromotor research, sports science and clinics.

## Figures and Tables

**Table 1:** Overview of results of the handgrip strength (*HS*) test stratified by school grade and hand preference. Higher values denote better results.

	Left-Preferent		Right-Preferent	
	Dominant	Non-Dominant	Dominant	Non-Dominant
**Ist grade**	11.35 (2.85)	10.40 (1.18)	10.60 (2.10)	10.20 (2.03)
**IInd grade**	11.85 (0.73)	12.30 (2.30)	12.10 (3.45)	11.70 (2.70)
**IVth grade**	14.85 (1.10)	15.75 (2.90)	15.80 (3.90)	15.10 (4.00)
**Vth grade**	15.80 (3.52)	14.80 (1.58)	17.90 (3.70)	17.20 (3.60)

Note. Data for each hand are presented in kilograms as median (interquartile range).

**Table 2:** Results of the *Floppy* test (time to completing) stratified by school grade and hand preference. Lower values denote better results.

	Left-Preferent		Right-Preferent	
	Dominant	Non-Dominant	Dominant	Non-Dominant
**Ist grade**	22.90 (2.43)	27.33 (10.26)	22.30 (4.58)	25.13 (5.08)
**IInd grade**	21.30 (2.97)	23.00 (4.73)	19.30 (3.92)	22.96 (5.05)
**IVth grade**	18.00 (3.43)	19.46 (1.85)	17.05 (3.19)	19.24 (4.05)
**Vth grade**	16.51 (3.79)	17.69 (1.73)	16.94 (3.20)	18.80 (4.16)

Note. Data for each hand are presented in seconds as median (interquartile range).

**Table 3:** Results of the *Thumb* test (time to completing) stratified by school grade and hand preference. Lower values denote better results.

	Left-Preferent		Right-Preferent	
	Dominant	Non-Dominant	Dominant	Non-Dominant
**Ist grade**	4.60 (1.49)	4.20 (1.30)	4.50 (1.31)	4.63 (1.53)
**IInd grade**	3.32 (0.69)	3.15 (0.90)	3.60 (1.13)	3.70 (1.10)
**IVth grade**	2.66 (0.49)	2.90 (0.61)	2.86 (0.90)	2.96 (0.88)
**Vth grade**	2.33 (0.20)	2.44 (0.60)	2.50 (0.66)	2.60 (0.76)

Note. Data for each hand are presented in seconds as median (interquartile range).

**Table 4:** Correlation matrix of motor tests stratified by hand preference. The sample size of right-handed children, as compared to left-handed ones, was considerably higher, biasing the statistical significance.

	Left-Preferent				Right-Preferent			
	LJ	4 × 10 SR	HS	Floppy	LJ	4 × 10 SR	HS	Floppy
**4 × 10 SR**	−0.272	—			−0.539 ***	—		
**HS**	−0.127	−0.554 **	—		0.433 ***	−0.276 ***	—	
**Floppy**	−0.387 *	0.123	−0.328	—	−0.171 **	0.218 ***	−0.512 ***	—
**Thumb**	−0.084	0.332	−0.348	0.348	−0.304 ***	0.361 ***	−0.564 ***	0.539 ***

Note. Data are presented as Spearman’s rho coefficients. LJ: long jump; HS: handgrip strength. 4 × 10 SR: 4 × 10 m shuttle run test. * *p* < 0.05, ** *p* < 0.01, *** *p* < 0.001.

## Supplementary Materials

The following are available online at https://www.mdpi.com/1660-4601/17/18/6705/s1: raw data.
